# Triaging Casual From Critical—Leveraging Machine Learning to Detect Self-Harm and Suicide Risks for Youth on Social Media: Algorithm Development and Validation Study

**DOI:** 10.2196/76051

**Published:** 2026-01-23

**Authors:** Sarvech Qadir, Ashwaq Alsoubai, Jinkyung Katie Park, Naima Samreen Ali, Munmun De Choudhury, Pamela Wisniewski

**Affiliations:** 1 Department of Computer Science Vanderbilt University Nashville, TN United States; 2 Department of Information Systems King Abdulaziz University Jeddah Saudi Arabia; 3 School of Computing Clemson University Clemson, SC United States; 4 School of Information University of Michigan Ann Arbor, MI United States; 5 School of Interactive Computing Georgia Institute of Technology Atlanta, GA United States; 6 International Computer Science Institute ICSI Berkeley, CA United States

**Keywords:** suicide/self-harm, machine learning, mental health, youth, natural language processing

## Abstract

**Background:**

This study aims to detect self-harm or suicide (SH-S) ideation language used by youth (aged 13-21 y) in their private Instagram (Meta) conversations. While automated mental health tools have shown promise, there remains a gap in understanding how nuanced youth language around SH-S can be effectively identified.

**Objective:**

Our work aimed to develop interpretable models that go beyond binary classification to recognize the spectrum of SH-S expressions.

**Methods:**

We analyzed a dataset of Instagram private conversations donated by youth. A range of traditional machine learning models (support vector machine, random forest, Naive Bayes, and extreme gradient boosting) and transformer-based architectures (Bidirectional Encoder Representations from Transformers and Distilled Bidirectional Encoder Representations from Transformers) were trained and evaluated. In addition to raw text, we incorporated contextual, psycholinguistic (linguistic injury word count), sentiment (Valence Aware Dictionary and Sentiment Reasoner), and lexical (term frequency–inverse document frequency) features to improve detection accuracy. We further explored how increasing conversational context—from message-level to subconversation level—affected model performance.

**Results:**

Distilled Bidirectional Encoder Representations from Transformers demonstrated a good performance in identifying the presence of SH-S behaviors within individual messages, achieving an accuracy of 99%. However, when tasked with a more fine-grained classification—differentiating among “self” (personal accounts of SH-S), “other” (references to SH-S experiences involving others), and “hyperbole” (sarcastic, humorous, or exaggerated mentions not indicative of genuine risk)—the model’s accuracy declined to 89%. Notably, by expanding the input window to include a broader conversational context, the model’s performance on these granular categories improved to 91%, highlighting the importance of contextual understanding when distinguishing between subtle variations in SH-S discourse.

**Conclusions:**

Our findings underscore the importance of designing SH-S automatic detection systems sensitive to the dynamic language of youth and social media. Contextual and sentiment-aware models improve detection and provide a nuanced understanding of SH-S risk expression. This research lays the foundation for developing inclusive and ethically grounded interventions, while also calling for future work to validate these models across platforms and populations.

## Introduction

### Background

Youth increasingly turn to social media platforms to express their feelings—even on highly sensitive topics such as self-harm or suicide (SH-S) [1-3]. These platforms provide an outlet where young individuals can seek support and solidarity by connecting with others who share similar struggles [4,5]. The anonymity and expansive reach of these networks allow users to express themselves more freely than in offline contexts; however, such openness can also expose them to potentially harmful content and triggering material that may exacerbate mental health issues [6]. In response, there have been growing efforts to predict SH-S behavior on social media using automated techniques [7,8]. The integration of machine learning (ML) and natural language processing (NLP) into mental health applications shows promising potential for early detection and intervention [9-11]. For instance, artificial intelligence (AI) chatbots implemented in schools to support student mental health have demonstrated how ML-powered technologies can address gaps where human counselors are unavailable [12]. As mental health challenges among youth rise, there is growing interest in using language as a window into psychological well-being. In online settings where traditional support structures may be absent, individuals often express distress through subtle shifts in everyday communication. NLP research in mental health has increasingly focused on identifying language-based signals of psychological distress in everyday communication. For instance, social media studies have shown that individuals experiencing depression or anxiety tend to use more first-person singular pronouns (eg, “I” and “me”), negative emotion words (eg, “sad” and “worthless”), and cognitive processing terms (eg, “think” and “understand”) [13]. Other research highlights that temporal focus—such as a shift toward past-tense verbs—and the use of absolutist language (eg, “always,” “nothing,” and “never”) are predictive of emotional dysregulation and suicidal ideation [14]. Individuals who frequently use absolutist terms may be more prone to anxiety and depression [15]. Additionally, research suggests that languages with obligatory future tense marking (eg, “will go” vs “go tomorrow”) are associated with lower national suicide rates, possibly by promoting a more future-oriented cognitive framework [16]. Beyond lexical cues, researchers have used syntactic complexity, semantic coherence, and sentiment trajectories to model psychological states across platforms like Reddit, Twitter (subsequently rebranded as X), and Tumblr [17]. However, when applied to the informal and unstructured language of social media, these systems face substantial challenges as youth often use dynamic slang, hyperbolic expressions, and indirect language cues [18-20], which can lead automated models to misinterpret benign or humorous exaggerations as genuine distress signals [21]. The resulting risk of overreaction or underreaction underscores the critical need for context-sensitive approaches that balance timely intervention with the preservation of safe expressive spaces [22-25].

In this work, we explore the effectiveness of combining traditional ML and transformer-based models (eg, Distilled Bidirectional Encoder Representations from Transformers [DistilBERT]), enriched with contextual, psycholinguistic, sentiment, and lexical features. A key innovation lies in our systematic evaluation of contextual window sizes, from single messages to extended subconversations, to determine how different levels of context affect model performance across nuanced SH-S categories: personal disclosures (“self”), references to others’ experiences (“other”), and nonserious, exaggerated mentions (“hyperbole”). We move beyond binary classification by systematically evaluating a 3-way schema “self,” “other,” and “hyperbole,” which captures subtle distinctions in SH-S expressions, with implications for more precise triaging. To our knowledge, this is the first study to analyze how varying context modeling strategies impact fine-grained SH-S classification performance using real-world, private youth data. By explicitly modeling the interplay between message content and its conversational context, we aim to develop more accurate, interpretable, and ethically grounded models for SH-S detection. These findings have direct implications for designing safer, context-aware digital interventions that preserve youth agencies while supporting mental health.

### Previous Work

Social media platforms are increasingly recognized as pivotal in identifying individuals exhibiting signs of SH-S [26]. Significant strides have been made by ML and social computing scholars in addressing mental health issues like SH-S through automated approaches [27]. Common practices in SH-S detection on social media include advanced ML and NLP techniques, such as Bidirectional Encoder Representations from Transformers (BERT)–based transformer architectures, which capture semantic meanings even when explicit keywords are absent, enabling the identification of at-risk individuals by detecting subtle linguistic markers associated with mental distress [8,27,28]. Despite these advances, such approaches have not been fully adapted to the specific language patterns used by youth on social media [29]. Youth share their experiences with SH-S in diverse ways, such as through hyperbolic expressions (without intent), discussions of others’ SH-S experiences, and explicit self-disclosures. A qualitative study by Ali et al [30] revealed that youth disclosures range from hyperbolic expressions (without intent) to discussions of others’ SH-S experiences and explicit self-disclosures. Furthermore, previous research has identified distinctions within SH-S, such as differentiating between suicidal ideation, the likelihood of a suicide attempt, and shifts toward suicidal ideation [7]. Most existing studies have employed binary classification methods for SH-S detection, failing to capture the multidimensionality of youth disclosures [31]. Given that online risks exist on a spectrum and can intensify over time, models need to differentiate among varying levels of risk rather than simply categorizing interactions as risky or not risky [32]. While substantial research has provided insights into SH-S prediction on public forums [33,34], there is limited understanding of how such risky discussions unfold in youth private discourses [29]. Our work addresses this multidimensional nature of youth SH-S disclosures in private conversation by performing multiclass classification. Furthermore, previous work identified a key limitation of existing SH-S detection systems is that, while they effectively capture textual content using features such as n-grams, bag-of-words, and deep learning–based word embeddings [35], they often overlook critical contextual factors and user characteristics, such as age and mental health history. This gap can lead to misclassifications that either trigger inappropriate interventions or miss genuine signals of distress [36,37]. To address these challenges, recent work has emphasized a human-centered machine learning approach, which integrates insights from individuals with lived experiences and those providing support to ensure that models are both accurate and interpretable [38]. Incorporating first-person annotations has proven particularly beneficial, as models trained on insider labels tend to outperform those based on third-party annotations [34,39,40]. Furthermore, transparent and explainable AI methods—such as attention-based models and Shapley Additive Explanations [41,42]—are being leveraged to highlight key features driving model decisions, thereby enhancing trust and accountability. Previous studies have shown that linguistic markers, such as absolutist terms, temporal focus, and future-oriented phrasing, are closely linked to mental health outcomes like depression, anxiety, and suicidal ideation, which underscore the value of subtle lexical and syntactic cues in predicting psychological risk [14]. Our work extends this line of research by exploring how such language patterns appear in youth social media conversations. Finally, integrating both linguistic and psychological signals, including sentiment shifts, interaction patterns, and user history [43], is crucial for accurately distinguishing between different forms of SH-S language, such as humorous references, self-disclosures, and discussions of others. By leveraging human-centered machine learning principles, we aim to develop a system that not only detects risk with high accuracy but also respects the agency and lived experiences of individuals expressing distress online.

### Goal of This Study and Research Questions

This study aims to develop a context-sensitive framework for detecting and classifying diverse types of SH-S language in youth private communications. Using data from the Instagram Data Donation (IGDD) project [44], where youth (aged 13-21 y) self-labeled their private direct messages as safe or unsafe, we build upon previous annotations of 2019 subconversations from 151 participants [30]. The annotated data are divided into three categories: (1) Self-disclosures of SH-S—explicit personal disclosures of SH-S ideation, (2) SH-S experiences of others—discussions involving SH-S incidents of others, and (3) hyperbolic representations of SH-S—metaphorical or humorous expressions of SH-S–related language.

The study is driven by 2 research questions (RQs). First, what is the best approach to distinguish between different types of SH-S language (humorous, disclosures about others, and self-disclosures) at the message and subconversation levels? Second, what are the contextual and linguistic characteristics that help distinguish between the 3 types of SH-S discourse?

To address these questions, we evaluated several ML models—including transformer-based architectures (BERT and DistilBERT) and classical classifiers (support vector machine [SVM], random forest, Naive Bayes, and extreme gradient boosting [XGBoost])—and enriched them with features such as contextual, psycholinguistic (linguistic injury word count [LIWC]), sentiment (Valence Aware Dictionary and Sentiment Reasoner), and lexical (term frequency–inverse document frequency) indicators. DistilBERT achieved the highest accuracy (99%) in distinguishing SH-S messages. Expanding the context from single messages to subconversations improved model accuracy to 91%. For RQ2, results highlighted that males tended to use hyperbolic SH-S language, females discussed others’ experiences, and nonbinary individuals more often shared personal SH-S experiences.

## Methods

This section outlines the dataset, preprocessing steps, feature engineering, classification models, and evaluation strategies used to address our research questions.

### Dataset

**Figure 1:**
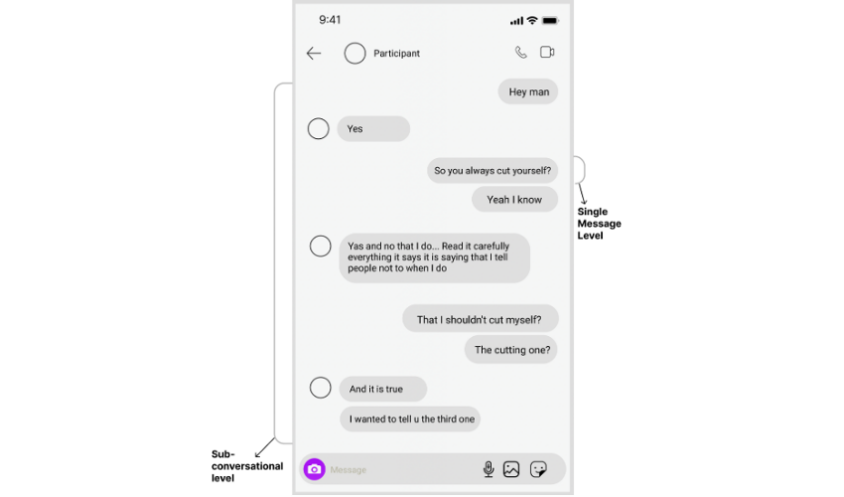
Message level and subconversational level.

We used a subset of the IGDD project dataset by Razi et al [44], specifically scoped and annotated by Ali et al [30]. In the study by Razi et al [44], researchers collected ecologically valid social media data through the IGDD project to study adolescent online safety. They recruited 195 English-speaking adolescents (aged 13-21 y) in the United States who had active Instagram (Meta) accounts during their teenage years and had experienced at least 2 direct message conversations that made them or someone else feel unsafe or uncomfortable. The dataset includes 32,055 private conversations, providing valuable insights into youth social media interactions and supporting ML models for online risk detection. Ali et al [30] manually annotated the original IGDD dataset for SH-S–related conversations, which resulted in 1224 SH-S–related conversations from 151 youth. The annotators had access to the entire conversation threads, and interrater reliability was calculated (Cohen κ=0.76) during the relevancy coding process to ensure consistency among the annotators in flagging the conversations as involving SH-S language. Interrater reliability was not calculated for the qualitative analyses because the thematic analysis process followed involved an inductive coding process where codes were developed and refined iteratively rather than applied from predetermined categories [45]. Since individual conversations could cover multiple topics, they further segmented them into distinct, topic-specific segments, referred to as subconversations. This process resulted in 2019 subconversations and 35,963 messages from the original 1224 SH-S–related conversations (Figure 1).

### Annotation Categories

Each subconversation in the SH-S dataset (n=2019) was annotated to capture context-specific interactions and categorized into 3 primary classes.

#### Self-Disclosures of SH-S

Instances where users explicitly disclose personal SH-S ideation (eg, “I’ve been cutting again. I don’t know how to stop.”). This category comprises 1262 (62.5%) subconversations and a total of 10,407 (28.9%) messages.

#### SH-S Experiences of Others

Conversations discussing SH-S incidents involving others (eg, “Did you know she cut themselves?”). This category includes 259 (12.8%) subconversations and a total of 7507 (20.9%) messages.

#### Hyperbolic Representations of SH-S

**Figure 2:**
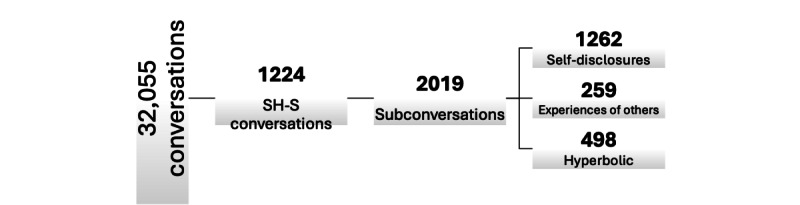
Overview of dataset distribution. SH-S: self-harm or suicide.

Instances where SH-S–related language is used humorously without serious intent (eg, “This homework is killing me!”). This category accounts for 498 (50.2%) subconversations and a total of 18,049 (35.7%) messages (Figure 2).

### Data Preprocessing

We applied a multistep preprocessing pipeline to clean the dataset while preserving essential linguistic cues.

1. Noise removal: Punctuation, hyperlinks, stop words, non-Latin words, and isolated numeric or single characters were removed. Emojis were converted to text using the Python demoji library to retain emotional cues.
2. Sensitive lexicon development: A lexicon of SH-S and violence-related keywords was constructed based on existing literature [46], guiding the filtering of messages containing SH-S disclosures.
3. Text embedding and filtering: Subconversations were embedded using Sentence-BERT (SBERT), and cosine similarity was computed against the sensitive lexicon. SBERT’s proven effectiveness in sentence-level retrieval and paraphrase detection [47] allowed us to identify indirect SH-S references.
4. Data augmentation: BertAug [48] was applied to increase the size and diversity of the training data by generating semantically coherent variations, thereby enhancing model robustness [49]

Table 1 represents the final number of subconversations for each category of SH-S.

**Table 1:** Number of subconversation instances before and after augmentation.

Category	Original instances, n	Post augmentation, n
Self-disclosures	498	996
SH-S^a^ experience of others	259	518
Hyperbolic representations	1262	2592
Total	2019	4106

^a^SH-S: self-harm or suicide.

The final dataset contained 4106 subconversations, offering a more balanced distribution across the 3 categories. To assess the impact of conversational context, we evaluated our best-performing model on the SH-S versus non–SH-S dataset at both the message-level (isolated messages) and the sub-conversational level (15-20 messages of holistic units). Previous research indicates that SH-S language is context dependent [50].

### Automatic SH-S Detection Approaches

To address RQ1, we implemented a diverse set of models:

- Transformer-based models: Fine-tuned pretrained BERT [51] and DistilBERT [52] models, leveraging their deep contextual understanding [53] and ability to capture subtle SH-S cues [54].
- Sequential models: Long short-term memory [55], convolutional neural network - bidirectional long short-term memory, and gated recurrent unit [56] architectures were used to model long-term dependencies and temporal patterns in subconversations.
- Classical ML Models: SVM, random forest, Naive Bayes, and XGBoost [57] were trained on engineered features to provide interpretable baselines [50].

### Analysis: Feature Engineering

For RQ2, we used a traditional non–end-to-end model (XGBoost, selected for its binary classification performance [57]) using features extracted from the annotated subconversations.

- Contextual features: Demographic data (ie, sex from IGDD survey responses) to explore influences on SH-S discourse.
- Psycholinguistic attributes: LIWC scores [58] covering affective processes (eg, sadness and anger), cognitive mechanisms (eg, causation and certainty), and social or personal concerns, normalized by subconversation length.
- Sentiment analysis: Polarity scores computed using Valence Aware Dictionary and Sentiment Reasoner [59] to capture the emotional tone.
- Lexical features: Term frequency–inverse document frequency [60] scores for the top 1000 terms to measure term importance.
- Additional linguistic scores: Toxicity, politeness, humor, empathy, and hate speech scores extracted using pretrained models from Hugging Face [61].

### Model Evaluation

Models were evaluated using standard metrics: accuracy, precision, recall, *F*_1_-score, area under the curve (AUC), and receiver operating characteristic curve. To mitigate overfitting, we used stratified k-fold cross-validation with k=5, which maintains the original class distribution across each fold. This method provided reliable performance estimates, especially in imbalanced datasets [62]. For deep learning models, validation sets were further used to apply early stopping based on validation loss to prevent overfitting [63]. Each model underwent hyperparameter optimization to maximize performance. For classical ML models (SVM, random forest, and XGBoost), hyperparameters (eg, SVM kernel type, XGBoost learning rate, max depth, and number of estimators) were optimized using grid search with cross-validation, which ensures that the hyperparameter evaluation is reliable and generalizable, while grid search exhaustively identifies the best combination of hyperparameters [64]. For deep learning models (long short-term memory, convolutional neural network - bidirectional long short-term memory, and gated recurrent unit), the hyperparameters—such as hidden layer sizes, dropout rates, learning rates, and batch sizes—were optimized using a random search combined with early stopping. For transformer models (BERT and DistilBERT), fine-tuning was performed with learning rate schedulers (eg, linear decay), with hyperparameters like learning rate, batch size, and warm-up steps optimized based on validation loss.

For RQ1, we fine-tuned DistilBERT (distilbert-base-uncased) separately for both message-level and snippet-level classification tasks. The dataset was split into 70% training, 10% validation, and 20% test, using stratified sampling to preserve class distributions across all splits. The test set was completely held out and used solely for final evaluation. All models were trained using the AdamW optimizer with a learning rate of 5e-5, batch size of 16, and a maximum input length of 512 tokens. We trained for up to 10 epochs with early stopping based on validation loss (patience=2) to prevent overfitting. For each classification task, we performed 5-fold cross-validation on the training set and reported average validation results across folds. To reduce overfitting and improve generalization, we applied data augmentation, including back-translation, synonym replacement, and paraphrasing techniques. For each class, we generated augmented samples to approximately double the training set size per class. Each original text was augmented up to 5 times, depending on the number of additional examples needed for that class. The augmenter used its default replacement probability of 0.3, substituting words with semantically appropriate synonyms. For back-translation, we translated text from English to German, and back to English, which added natural lexical and syntactic variation while preserving meaning. We further ensured model stability through a power analysis [65] to confirm that the training data size was sufficient to detect performance differences across model variants with acceptable statistical power. The reported performance metrics reflect results on a completely held-out test set and were consistent across folds and evaluation phases, supporting model generalization and robustness.

### Unpacking Results Qualitatively

To gain deeper insights beyond quantitative metrics, we conducted a qualitative reading [35] focused on unpacking model misclassifications, feature importance, and demographic influences on SH-S discourse. First, we examined instances where models failed at the message level but succeeded at the subconversational level in RQ1. Second, unpacking psycholinguistic feature contributions and additional features. We conducted a detailed analysis of the LIWC features [58], evaluating the influence of its 64 categories on model predictions. This involved both quantitative and qualitative steps, such as using Shapley Additive Explanations values, and we identified which LIWC categories most significantly influenced the model’s classification decisions. To validate these findings, we manually reviewed subconversations with high activations in key LIWC categories. Furthermore, to understand the significance of each of the additional linguistic features, that is, toxicity [66], politeness, humor, empathy, and hate speech [67], we used ANOVA tests [68] to help understand if there was any significance in prediction. Finally, we conducted demographic analysis (sex and discourse types). To provide additional context on the dataset, we include demographic statistics from the IGDD survey responses, which describe the characteristics of the 151 youth participants whose conversations were analyzed in this study. Most participants identified as female (n=107, 71%), followed by male (n=30, 20%), and nonbinary or preferred not to self-identify (n=14, 9%). For RQ2, we also explored potential demographic influences on SH-S discourse. We conducted a chi-square [69] to examine the relationship between sex and different types of discourse (self-disclosure, SH-S experiences of others, and hyperbolic representations). The test revealed statistically significant differences (*P*<.05) in discourse types across sex groups.

### Ethical Considerations

The secondary analysis of this dataset was reviewed and approved by the Vanderbilt University Institutional Review Board (IRB #222197). The original user study in which the data were collected was approved by the University of Central Florida Institutional Review Board (IRB #00001136). In the original study, informed consent was obtained from all participants; for any participants who were minors, consent or assent procedures were completed in accordance with the approving ethics board requirements. Participants in the IGDD study were compensated with a US$50 Amazon gift card for their time and data contribution. All team members who accessed the data completed required human subjects protections (Collaborative Institutional Training Initiative) and Protection of Minors training before working with the dataset. Moreover, we had a child abuse and imminent risk reporting protocol in place, and any concerning posts were reviewed by the Director of Risk Management and Child Protection to ensure that we met mandated reporting requirements. To protect participant privacy, all data were deidentified during the scoping process. In addition, any quotations included in this manuscript have been edited to remove or mask names, locations, and other potentially personally identifiable details while preserving their substantive meaning.

## Results

### Overview

In this section, we first present the results of the classifiers that predict SH-S risks at the message level (ie, binary classification). The classifiers were further evaluated with the best-performing end-to-end model at the message and subconversational level (RQ1). Next, we added different features to the best non–end-to-end model (RQ2), and the results were presented, highlighting the unpacking of contextual analysis of our model.

### Automated Classification of SH-S in Private Conversations of Youth (RQ1)

#### Binary Classification (Message Level)

For the classification task to identify SH-S versus non–SH-S messages (binary classification), the DistilBERT (end-to-end learner; accuracy=0.99, precision=0.99, *F*_1_-score=0.99, recall=0.98, and AUC=0.99) model outperformed the other models used across all assessed metrics (Table 2).

**Table 2:** Performance metrics of various models in binary classification with non–self-harm or suicide data, categorized as end-to-end learners or non–end-to-end learners.

Models	Accuracy	Precision	Recall	*F*_1_-score	AUC^a^	Type
Random forest	0.85	0.88	0.73	0.80	0.83	Non-E2E^b^
SVM^c^	0.89	0.88	0.84	0.86	0.88	Non-E2E
Naive Bayes	0.80	0.87	0.57	0.69	0.91	Non-E2E
XGBoost^d^	0.94	0.91	0.94	0.93	0.98	Non-E2E
CNN^e^	0.88	0.88	0.83	0.85	0.95	E2E
CNN-BiLSTM^f^	0.89	0.85	0.88	0.87	0.90	E2E
LSTM^g^	0.85	0.83	0.79	0.81	0.90	E2E
GRU^h^	0.84	0.82	0.78	0.80	0.92	E2E
BERT^i^	0.97	0.92	0.98	0.95	0.99	E2E
DistilBERT^j^	0.99	0.99	0.98	0.99	0.99	E2E

^a^AUC: area under the curve.

^b^E2E: end-to-end.

^c^SVM: support vector machine.

^d^XGBoost: extreme gradient boosting.

^e^CNN: convolutional neural network.

^f^BiLSTM: bidirectional long short-term memory.

^g^LSTM: long short-term memory.

^h^GRU: gated recurrent unit.

^i^BERT: Bidirectional Encoder Representations from Transformers.

^j^DistilBERT: Distilled Bidirectional Encoder Representations from Transformers.

This high level of accuracy was maintained even when keywords associated with SH-S were removed from the dataset. The results from the keyword removal process using SBERT, which was a methodological approach, also indicated a notable performance with the DistilBERT model, achieving high metrics: accuracy at 0.88, *F*_1_-score at 0.87, precision at 0.80, recall at 0.95, and an AUC of 0.94.

#### 3-Class Classification (Message and Subconversational Level)

Next, we compared the DistilBERT model’s performance on a single message and a subconversational level. As seen from Table 3, the inclusion of context at the subconversational level resulted in a marked improvement in the model’s performance, with all the accuracy metrics showing an increase.

**Table 3:** Performance metrics at the message and subconversation levels.

Level		Precision	Recall	*F*_1_-score	Accuracy
**Message**					0.89
	Overall	0.89	0.89	0.89	
	Hyperbole	0.90	0.97	0.94	
	Other	0.86	0.78	0.82	
	Self	0.86	0.73	0.79	
**Subconversation**					0.91
	Overall	0.91	0.91	0.91	
	Hyperbole	0.94	0.94	0.94	
	Other	0.90	0.95	0.92	
	Self	0.83	0.79	0.81	

Our qualitative analysis of classification results confirmed that end-to-end models like DistilBERT, which learn to predict directly from raw text to classification labels, benefited from larger context windows. Specifically, we observed an overall improvement across all performance metrics when moving from message-level to subconversation-level classification. The overall accuracy increased from 0.89 to 0.91, reflecting more accurate predictions with a broader conversational context. This trend was even more pronounced within individual classes. For the “other” class, *F*_1_-score improved from 0.82 to 0.92, and for the “self” class, from 0.79 to 0.81. The model was unable to determine the true intent behind potentially alarming situations when analyzing single messages in isolation. Specifically, the model initially categorized self-disclosure messages indicating imminent risk as hyperbole, likely due to the common use of exaggerated language in casual conversations, particularly among youth. However, responses from conversation partners often provided critical interpretive signals that helped the model correct its classification when additional context was introduced.

For example, in the following subconversation, the model initially classified the message “*And want to kill myself*” as hyperbole risk. Yet, when the model processed the entire subconversation, it identified contextual cues such as “*No don’t kill yourself*” indicating that the situation was serious rather than exaggerated and highlighting an ongoing struggle and the urgency of the distress being expressed.

P: And want to kill myself

O: I can’t believe you have to deal with this everyday

O: No don’t kill yourself, It’s not a good idea

P: I’m killing myself

For 2-way conversations, “P” refers to the primary participant, whose conversation is being analyzed. “O” denotes the other individual involved in the exchange. For group conversations, we appended numbers “O1,” “O2,” “O3,” and so on, to denote the other individuals participating in the group conversation. Conversely, the opposite scenario occurred when the model misclassified messages as immediate “self” risk. Upon further examination of the context, it became clear that the youth was either exaggerating their emotions or discussing the struggles of someone else. This was likely because the messages appeared to directly address an individual who may be struggling with suicidal thoughts or because they contained words like “cut” and “I,” which are often associated with personal distress and self-harm. The pronoun “I” typically signals self-referential language, which, when combined with distress-related terms, may lead the model to interpret the message as expressing suicidal intent. However, additional context, such as the presence of laughter, such as “HAHAHAHAHA,” provided crucial disambiguation, shifting the interpretation toward humor or hyperbole. In the following subconversation, the initial classification of self-risk was corrected when the broader exchange revealed that the discussion was playful rather than an actual self-harm disclosure:

O: Can you tell me The frick, Did you just cut, HAHAHAHAHA

P: It’s my middle

O: I DON’T CARE, WHYD YOU CUT, NOO, Are you kidding me. COME ON,

WIPE IT UP, And I’ll tell you, And take a picture of it cleaned

O:Put a bandaid on your cut

P: I didn’t cut myself, It was food I KNEW IT. I knew I used a different finger to draw red

In addition, when additional context from the broader subconversation was added, it became evident that the participants were not discussing their own distress but rather engaging in third-person discussions about distressing events. For example, in the following subconversation, the message was initially classified as “self,” but with added context, it became clear that the participants were reacting to the news of someone else’s passing.

P: How are you feeling

O: Still in shock but, life goes on. Gotta keep moving forward

P: And you should know, suicide is not the answer, think about the good things in life

O: Yeah, you have to try and find peace

P: Yeah I couldn’t believe it. This is a sad news

O: May he rest in peace. I knew this guy

The improved performance at the subconversational level demonstrates the benefit of incorporating context, providing richer semantic information than isolated messages.

Table 4 below presents a qualitative breakdown of frequent misclassifications. The most common confusion occurred between self-disclosure and hyperbolic expression, particularly when tone was ambiguous or sarcasm was present. Other frequent errors stemmed from third-person framing, generalizations, or lack of conversational context, underscoring the limitations of message-level models.

**Table 4:** Examples of misclassifications between message and subconversational levels.

Misclassified type	Misclassified cases (actual), n (%)	Example message	Example subconversation	Likely cause
Other to self	5 (6.02)	Who repeatedly threatened suicide and harassed her if she didn’t spend every single free moment talking to him?	Have you ever seen [person’] video on her ex boyfriend? Who repeatedly threatened suicide and harassed her if she didn’t spend every single free moment talking to him? She met him in person and thought she was safe because he was a scrawny nerd and thought “I can totally fight him if he forces himself on me.” She couldn’t defend herself when she really needed it because she had been too mentally worn down by her boyfriend to resist and froze.”	Third-person narrative was judged as suicide due to lack of context
Hyperbole to self	15 (18.07)	guys i’m being stupid bc ive continued to snap thomas and like minutes ago he snapped me “you cute :)” and i literally wanna kms i’m disappointed in myself also to add insult to injury he was in his boy scount uniform	guys i’m being stupid bc ive continued to snap thomas and like minutes ago he snapped me “you cute :)” and i literally wanna kms i’m disappointed in myself also to add insult to injury he was in his boy scount ahhhhdamnnn you got a boy scout noooo he’s not my lol you right he’s your boy	Sarcasm, dramatic and violent tone interpreted literally as personal disclosure
Hyperbole to other	2 (2.41)	She is like,, talking to everyone in the groupchat and just like about to die lol	“I do need to get over this disgusting possessiveness but it could be good! not weird! And I’m not gonna private lol that’s weirddddd Not flirty! She is just talking normally but my possessiveness is getting BAD how was your orientation tho!!! she prolly just has a flirty personality tho which sucks i think you should talk to her in private message!!! lmao Well I have job orientation so I couldn’t join lmoa They voice chatted w/o me too : She is like,, talking to everyone in the groupchat and just like about to die lol My jealousy/possessiveness is getting REALLY bad lol”	Ambiguous intent; informal tone predicted as someone else’s disclosure
Other to hyperbole	15 (18.07)	Wow but u up future [me] to kill myself	P: [‘Tomorrow! Guess I ’ ll keep him. .. O: lol I cannotttttttt P: Exactly O: ooooh dad Meee P: Wow but u up future [me] to kill myself P: Omg describes me still to dry out feet Ok [person]]	Figurative expressions misread as hyperbole
Self to other	7 (8.43)	my friend sent me a suicide message	P: and she wasn’t responding for at least minutes to an hour P: my friend sent me a suicide message- O: that’s a lot of chat to read O: lemme read the chat O: wait? what happened? P: she texted me, luckily she’s still here but she barely has the strength to keep moving O: I had to get the sword out just to use it to reach a pair of scissors I accidentally got stuck somewhere-	Third-person framing; lack of first-person indicators in the message level for prediction of Self category. Expanded context in sub-conversational level
Self to hyperbole	39 (46.99)	I did and I’m gonna continue it bc it won’t let me die this week next week I’ll continue to be dead	P: “I’d rethink my life choices My childhood Wellfuq private O: Ill join P: I did and I’m gonna continue it bc it won’t let me die this week next week I’ll continue to be dead O: I CRI ERYTIME FAM I THOT U ENDED URSRLF P: YESSS P: I literally thought i was going to get shit done but then i realized we’ve been blessed with the internet”	Overlapping emotional expression and slang

### Contextual and Psycholinguistic Characteristics of Youth SH-S Conversation (RQ2): Psycholinguistic Characteristics of Youth SH-S Conversation

We further unpacked the linguistic features and presented the results from a qualitative analysis evaluating how the psycholinguistic characteristics were associated with classification results for each category (self, other, and hyperbole). We completed this analysis at the subconversation level rather than the message level due to more context, as confirmed by RQ1 results. Our results show that psycholinguistic words in each subconversation helped predict each category. For example, the words associated with tentativeness (“tentat”) showed high importance for prediction in the “self” category. This suggested that individuals speaking from personal experience expressed uncertainty or hesitation, which could reflect the complexity of their feelings or situations. This was seen in the use of words like “maybe,” “or,” “anyone,” “if,” and “anything.” For example, in the following conversation, tentative words helped with the prediction of a participant reminiscing about something from their past:

P: i was all like “oh man what if this all gets so bad that i like self harm or something i’m going to cry” because i wanted attention and then fifth grade came and i was just fine for no reason

O: haha so i remember i was like “if i don’t get this many likes in this many days i’m leaving”

P: i remember posting this thing about how i was all “depressed”

Furthermore, words associated with space, such as “spatial” and “time” language, were prominent for the prediction of the “hyperbole” category, which implied a discussion about SH-S that involves words related to physical spaces or distances. This may be relevant in conversations where individuals are talking about other people or situations removed from themselves. For example, in the following subconversation, spatial terminology played a crucial role in amplifying the description of a strenuous physical routine. The words underlining physical locations and movements, such as “in the heat,” “up and down hills,” “on our track,” “on the hot hard ground,” and “on the track,” constructed a scenario rife with exertion. The hyperbolic essence is further enhanced by the repetitive mention of these terms, intensifying the narrative. The speaker used this spatial language to dramatically overstate and exaggerate, typical of hyperbolic discourses. For instance, phrases like “weird ass suicides on the track” and the exaggerated claim of being ready to “pass out” further illustrated the intensity of the workout for dramatic effect.

*O1: nahhh we be running with full pads in the heat up and down hills and the sun shine directly* *on our track*

O2: in the afternoon we run a mile do drills where we gotta lay down on the hot hard ground

O3: we do bleachers we do laps if our group came in last we do mad push ups crutches etc.. we do weird ass suicides on the track.

In addition, language focused on family-related words (“home”) and social processes (“social”) aided in the prediction of the “other” category, which implied that discussions about others’ situations in a home or family context or interactions when discussing others’ experiences with SH-S. For instance, the following conversation talks about how social and family keywords, that is, “roommate,” “person,” “person1,” “names,” helped in prediction to models when talking about the experiences of others.

P: The one that never showers has a new kid roommate and everyone in the suite beside her is new. Besides some that had accidents the rest r ppl that no one wanted to room with.

O: Most returners with new kid roommates? Watch out.

P: Yeahhhhhh. And it makes [person] hecka sad and he’s been staying in my room with me cause it’s too much for him.

O: Cause [person] has him and he’s dying cause [person1] won’t talk about anything except suicide and gays.

P: At least his room is clean and he showers better than some annoying ppl.

As such, the psycholinguistic features aided the model in prediction based on the higher frequency of specific features in individual categories.

### Identifying Key Differentiators in SH-S Conversations

To evaluate the role of additional linguistic features in distinguishing between categories (hyperbole or humor, other, and self), a 1-way ANOVA test was conducted. Table 5 presents the results of the ANOVA analysis.

**Table 5:** ANOVA results for distinguishing conversational categories. Statistically significant features (P<.05) indicate meaningful variations across categories.

Feature	*F* test (*df*)	*P* value
EmpathyScore	369.22 (2, 2141)	<.001
Toxicity	144.63 (2, 2141)	<.001
PolitenessScore	22.64 (2, 2141)	<.001
HumorScore	18.74 (2, 2141)	<.001
HateSpeechScore	1.14 (2, 2141)	.32 (ns^a^)

^a^ns: not significant.

Among the features analyzed, EmpathyScore (*P*<*.*001) was the strongest differentiator, particularly in *self* messages. This suggests that self-harm disclosures often contain highly empathetic language, either from the individual expressing distress or from responders offering support.

P: It’s not very good please don’t get mad. It’s how I calm myself. It helpss.

O: Oh, I am not mad. What matters to me right now is that you’re feeling better...Though, is it okay if I request that we attempt to find a safer method in the future? There are endless ways, we can find one!

Here, the self message contains hesitation and a plea for nonjudgment, while the response demonstrates high empathy by validating the user’s feelings before gently encouraging an alternative. These findings reinforce the importance of empathy detection in SH-S identification—messages with high “EmpathyScore” often signal distress and should be carefully analyzed rather than dismissed as neutral or unimportant. Additionally, toxicity (*P*<*.*01) was more prevalent in *hyperbole or humor* messages, indicating that sarcasm and exaggeration often introduce aggressive or negative wording. However, this poses a challenge for SH-S detection, as some genuine disclosures may also be flagged as toxic despite containing cries for help.

P: I sent this to her and said you included [person] before meeee Fucking kill me, LOL legit and we will always be novices in their eyes even when we are not.

Here, the phrase “*Fucking kill me*” demonstrates self-harm references with violent references increasing the overall toxicity of the subconversation. Interestingly, toxicity and EmpathyScore were positively correlated (*r*=0*.*41), highlighting a key challenge identified in content moderation systems; messages flagged as toxic also contain empathy. This indicates that toxicity does not always equate to harmful intent—rather, some individuals express distress in a way that appears toxic but is a plea for help.

P: he would call me a fat whore and a bitch, and would tell me to kill myself, and i stayed with him for six months and cried everyday.

In this message, the high toxicity score is due to the offensive language describing verbal abuse, yet the high empathy score reflects the emotional weight of the experience being shared. If an AI system were to automatically filter high-toxicity messages as humor, it might wrongly remove important self-harm disclosures, preventing individuals from receiving support. Therefore, there is a need for context-aware, explainable moderation rather than strict toxicity-based filtering.

### Contextual Characteristics of Youth SH-S Conversation

**Figure 3:**
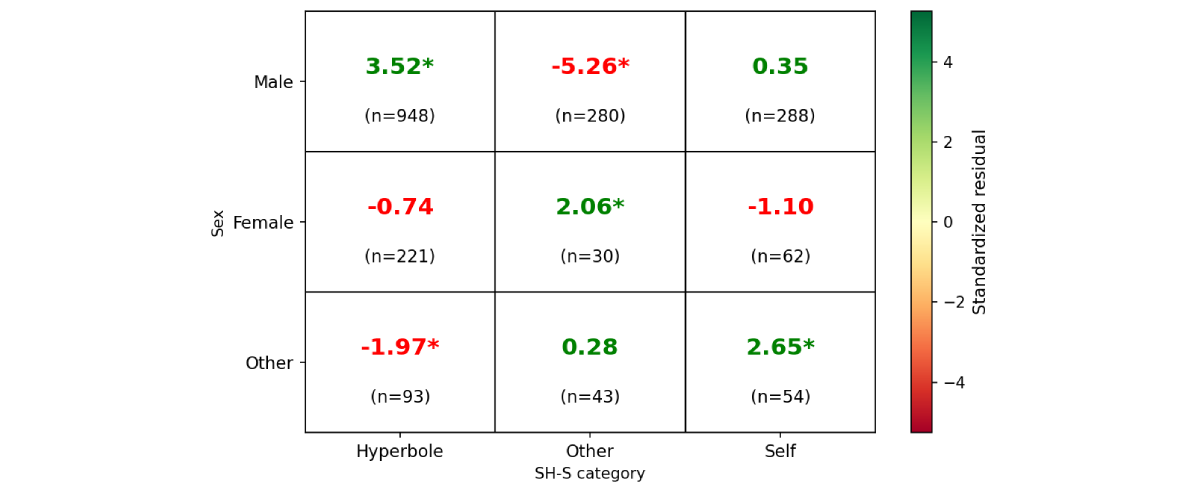
Residuals: sex versus category. The magnitude of the standardized residual (eg, above 2 or below –2) indicates the strength of this deviation (|z|>1.96; *P*<.05), denoted with (*) with the value. SH-S: self-harm or suicide.

Next, we unpacked the contextual factors and presented the results from the relationship between “sex” feature and discourse categories (“hyperbole,” “other,” and “self,”) as captured in Figure 3, highlighting noteworthy patterns in the communication styles among different sexes using a chi-square test. In this analysis, standardized residuals with an absolute value greater than 1.96 (indicated by an asterisk) denote a significant deviation from expected frequencies (*P*<.05).

To examine sex-specific patterns in SH-S discourse, we conducted a chi-squared test of independence between sex identity (female, male, and other) and SH-S discourse categories (hyperbole, other, and self). The analysis yielded a statistically significant association (*χ*²_4_=57.16; n=2019; *P*<.001), indicating that the distribution of SH-S expression types significantly varied by sex. Standardized residuals revealed notable patterns in language use across sex groups (Figure 3). Male participants were substantially more likely than expected to use hyperbolic SH-S language (residual=3.52*), reflecting a frequent use of exaggerated or nonliteral references to SH-S. At the same time, they were significantly less likely to discuss the SH-S experiences of others (residual=–5.26*), suggesting that third-person narratives were underrepresented in male-authored conversations. Participants identifying as nonbinary exhibited a distinct linguistic profile. They were less likely to use hyperbole (residual=–1.97) and more likely to disclose personal SH-S experiences (residual=2.65*), reflecting a stronger inclination toward emotionally vulnerable and direct expression of mental health challenges. Female participants, on the other hand, were more likely to reference others’ SH-S experiences (residual=2.06*), indicating a tendency to discuss SH-S in the context of third parties, such as in gossiping situations.

## Discussion

### Principal Findings

Prior studies have typically focused on binary classification of SH-S or used public social media datasets. In contrast, our work introduces a granular classification of SH-S disclosures. We further examine how varying levels of conversational context influence model performance and incorporate psycholinguistic and contextual signals into transformer-based models to improve interpretability. Our study highlights the effectiveness of using transformer-based models, particularly DistilBERT, for detecting SH-S ideation in youth’s private Instagram conversations. DistilBERT achieved 99% accuracy in binary SH-S classification and 91% accuracy in multiclass categorization when extended to subconversational context. Incorporating features, such as sentiment, psycholinguistic cues, and contextual windows, significantly improved model performance. We also uncovered meaningful sex-based patterns in SH-S language: males were more likely to use hyperbolic expressions, females tended to discuss others’ experiences, and nonbinary individuals predominantly shared personal disclosures.

### The Importance of Context in Detecting SH-S

The classification findings (RQ1) highlight the importance of using our context-sensitive approach, which addresses the limitations of traditional binary classification and the reliance on single messages that constrained the model’s ability to capture the nuanced meanings and intentions of SH-S discourses. This approach enabled us to develop an automatic detection model that distinguished between casual expressions and critical SH-S situations, achieving an accuracy of 91%. Although this level of accuracy is noteworthy, we advocate for a paradigm shift in future research. Rather than focusing solely on improving model accuracy or optimizing algorithms [70], greater emphasis should be placed on identifying and understanding the nuanced variations within SH-S–related content, as well as determining the optimal conversational context necessary to inform and enhance the detection capabilities. By incorporating these dimensions into detection models, researchers could differentiate low-risk expressions from those that signal imminent danger and reduce both false negatives, where serious risk may be overlooked, and false positives, where benign expressions are mistakenly flagged, as we showed in our error analysis. Recognizing these distinctions supports more targeted and ethically responsible interventions, ensuring that individuals in genuine need receive appropriate care while minimizing unnecessary responses. Future models could advance this framework by adopting more refined annotation schemes, as previous work has identified various proxies indicative of mental health challenges online. For example, online harassment has been shown to precede declines in mental health [71], and groups of teens who engaged in self-harm offline were found to participate in high-risk sexual conversations with strangers online [72]. These findings, along with the granular approach used in this study, underscore the complexity of identifying SH-S cases in online conversations, far beyond what a simple binary classification can capture. In addition, we recommend future research to leverage advanced techniques, such as reinforcement learning [73], to dynamically identify and integrate the most informative conversational context. Such efforts will advance the field toward a more nuanced, context-aware, and human-centered approach to SH-S detection across both clinical and online environments.

### Contextual and Linguistic Implications for Automated SH-S Detection

The prominence of tentative words (eg, “maybe,” “if,” “anyone,” and “anything”) in SH-S ideation disclosures suggests that individuals may exhibit uncertainty or hesitation when expressing their personal experiences [74]. This observation aligns with previous psychological research, which indicates that ambivalence is common among individuals contemplating SH-S, reflecting an internal struggle or emotional distress [75]. Consequently, AI models for SH-S detection must be designed to contextualize such uncertainty rather than dismiss it outright. Flagging potential risk cases based solely on tentative language may lead to overlooking individuals in distress; therefore, future work should consider incorporating longitudinal analyses to determine whether repeated use of tentative expressions correlates with escalating distress over time [76]. Furthermore, the model’s reliance on spatial references (eg, “on the track” and “up and down hills”) and temporal references (eg, “in the heat”) for classifying hyperbolic language highlights the role of vivid, metaphorical descriptions in exaggerated narratives. In social media conversations, hyperbole is frequently used as a form of dark humor, exaggeration, or emphasis rather than as an indication of genuine self-harm intent [30]. Previous studies have demonstrated that language not only conveys information but also shapes cognitive processes; for example, spatial and temporal metaphors influence how individuals conceptualize and articulate their experiences [77]. Given the prevalence of such hyperbolic expressions among youth, detection systems must adapt to evolving linguistic trends. The integration of sentiment analysis and contextual embeddings is recommended to enhance classification accuracy by distinguishing between distress-driven hyperbole and casual or humorous exaggeration. The frequent mention of social and familial words (eg, “roommate,” “person,” and “home”) suggests that discussions regarding others’ SH-S experiences are often framed within interpersonal and domestic contexts. This linguistic pattern reflects how individuals process and externalize their concerns by embedding them in familiar social environments [78]. Research indicates that discussing traumatic events, such as another’s SH-S, can serve as a coping mechanism, helping individuals process their emotions through interpersonal narratives [79]. For automated detection systems, it is critical to differentiate between self-reports and third-party observations. While discussions about others may not signal personal risk, they can indicate a user’s concern for someone at risk [80]. Future research should explore networked conversational analysis to identify patterns in how information about SH-S is shared, potentially guiding the design of interventions that support indirect reporting in real-time crisis situations.

### Sex-Based Patterns in SH-S Detection

Our analysis reveals distinct sex-based patterns in SH-S communications. Specifically, males in our dataset more frequently used hyperbolic or exaggerated language, females tended to discuss SH-S in the context of others’ experiences, and nonbinary youth were more likely to share personal self-disclosures. One potential explanation is that these differences mirror varying psychological needs and coping mechanisms, where males are using violent language [81]. For example, males may use humor or hyperbole as a form of emotional regulation or deflection [82], while females may rely more on social or communal support, thus centering their discussions on peers, friends, or family members [83]. Meanwhile, nonbinary youth could find direct self-disclosure to be a more authentic way to articulate distress, potentially reflecting lived experiences tied to identity-related stressors [84]. Individual communication styles are shaped by intersecting factors, such as cultural background, social context, and personal history, which means no single sex group is homogeneous in its expression of SH-S [85]. Additionally, differences in disclosure may stem from how comfortable individuals feel discussing mental health in private online spaces, rather than from any inherent sex-based communication pattern [86]. Future research should therefore investigate not only what different sex groups share online but also the broader context—how, when, and why they choose to share. An intersectional approach that considers overlapping identities (eg, race, ethnicity, and sexual orientation) may offer deeper insights and help prevent models from misclassifying or overlooking at-risk behaviors. Furthermore, detection systems need to account for these diverse linguistic and psychological signals without reinforcing biases. By doing so, we can develop more inclusive, accurate, and ethically grounded tools for identifying and responding to SH-S discourse across varying demographic groups.

### Toward Clinical and Educational Applications

While our study primarily focuses on advancing SH-S detection models through nuanced classification and context-aware techniques, our findings lay important groundwork for translation into real-world clinical and educational settings. One of the major challenges for automated risk detection systems is the high rate of false positives [87], which can overburden already stretched mental health infrastructures and erode trust among stakeholders [88]. Our approach can help address this concern by enabling triaged classification systems (ie, differentiating low-risk, hyperbolic expressions from high-risk, and self-disclosure content) that prioritize urgency and minimize unnecessary escalations. For instance, high-confidence “self” messages may be routed for review by school counselors or clinical professionals, while “other” or “hyperbole” cases can prompt peer-based support, reflective check-ins, or educational messaging. These differentiated outputs could be integrated into existing digital mental health infrastructures such as Crisis Text Line [89], Kognito simulations [90], or school-based early warning systems to assist professionals in identifying emerging risks. Rather than triggering blanket alerts, the model output can inform tiered intervention protocols that optimize limited human resources and reduce unnecessary escalations [91]. Importantly, such integration should not replace human support but rather augment it by flagging concerning patterns at scale while preserving user autonomy and privacy. Furthermore, schools are well-positioned to implement suicide prevention strategies [92] but often rely on direct disclosures, missing the indirect language youth use online [93]. Our work suggests that expanding these systems to include models trained on authentic youth expressions, including hyperbole and third-person narratives, could reduce false negatives and better capture subtle signs of distress. Beyond detection, insights from our findings have important implications for mental health education. In school-based programs, educators can incorporate reflective writing or discussion-based activities using examples of SH-S–related messages to help students explore how emotions are expressed online. For instance, educators can teach youth how to express emotional distress more descriptively to reduce misinterpretation and unintentional triggering. In addition, our findings highlight the need to equip youth with the skills to recognize when peers might be signaling distress, directly or indirectly. These educational opportunities together can help youth not only to seek help when needed but also to support one another in navigating emotionally charged conversations in safe and constructive ways.

### Limitations and Future Work

A primary limitation of our study is the reliance on human annotations from Ali et al [30], where the instances analyzed were not flagged by the target participants themselves but were instead annotated by researchers. Additionally, the challenges of obtaining datasets in our domain may limit the generalizability of our findings to other platforms. Another limitation is that, due to the sensitive nature of the data, the original researchers have only shared it on a limited basis with trusted collaborators. This restricted access means that the dataset cannot be made publicly available, thus limiting opportunities for independent validation and replication of our findings. Another limitation identified in our error analysis was that existing models, like DistilBERT, do not explicitly account for slang and may struggle with the informal language used by youth. While some slang and code words, such as “kms” or “dies,” were captured due to their frequency in the dataset and contextual embeddings, less common or emerging terms may still be missed. In addition, our model was trained exclusively on English language data from US-based adolescents, which may limit its applicability to non-English speaking or culturally distinct youth populations.

Future studies should apply our approach to other youth datasets where the data is flagged either by the participants themselves or by clinicians. This would help ensure that the models are responsive to the subjective experiences of individuals at risk. Secondly, incorporating insights from clinical experts in reviewing flagged data could provide a more comprehensive understanding of risk levels and improve the classifiers’ accuracy. Further work should focus on enhancing ML models by integrating slang and other colloquial expressions to improve their accuracy in detecting SH-S language. Deeper attention should be paid to the detection of evolving code word conversations used by youth to talk about distress in indirect ways. The use of mutually exclusive labels (“self,” “other,” and “hyperbole”) may oversimplify complex expressions that span multiple categories. Future work could explore more flexible annotation schemes—such as multilabel classification or probabilistic tagging—to better capture the fluidity of online discourse. Additionally, incorporating human-in-the-loop testing or clinician usability assessments would offer valuable insight into deployment challenges and model effectiveness in real-world settings. Finally, researchers should work on assessing the timeliness of detection, ensuring that the models classify risks accurately in a time-sensitive manner. Future extensions should also consider applying this work to other languages (eg, Spanish and Chinese) and regions (eg, Asia and Europe) to explore how cultural semantics shape youth expressions of self-harm, sarcasm, and distress.

### Conclusion

This study demonstrates the potential of ML models—particularly transformer-based architectures like DistilBERT—to accurately detect SH-S ideation within youth’s private social media conversations. By moving beyond binary classification and incorporating contextual, psycholinguistic, sentiment, and lexical features, our approach captures the nuanced spectrum of SH-S expressions, from hyperbole to personal disclosures. Importantly, we find that expanding the context window to subconversations significantly improves classification accuracy, underscoring the critical role of conversational context in understanding youth mental health language. Furthermore, sex-specific patterns in SH-S expression highlight the need for inclusive models that account for diverse linguistic behaviors. As digital platforms become central to youth communication, our findings emphasize the importance of context-aware, ethically designed interventions that can support timely and sensitive mental health responses.

## Abbreviations

AI: artificial intelligence
AUC: area under the curve
BERT: Bidirectional Encoder Representations from Transformers
DistilBERT: Distilled Bidirectional Encoder Representations from Transformers
IGDD: Instagram Data Donation
LIWC: linguistic injury word count
ML: machine learning
NLP: natural language processing
RQ: research question
SBERT: Sentence-Bidirectional Encoder Representations from Transformers
SH-S: self-harm and suicide
SVM: support vector machine
XGBoost: extreme gradient boosting
